# Trends and disparities in avoidable, treatable, and preventable mortalities in South Korea, 2001-2020: comparison of capital and non-capital areas

**DOI:** 10.4178/epih.e2022067

**Published:** 2022-08-16

**Authors:** Sang Jun Eun

**Affiliations:** Department of Preventive Medicine, Chungnam National University College of Medicine, Daejeon, Korea

**Keywords:** Mortality, Health inequities, Health status disparities, Healthcare disparities, Trends, Seoul, Republic of Korea

## Abstract

**OBJECTIVES:**

This study aimed to describe the regional avoidable mortality trends in Korea and examine the trends in avoidable mortality disparities between the Seoul Capital Area and non-Seoul-Capital areas, thereby exploring the underlying reasons for the trend changes.

**METHODS:**

Age-standardized mortality rates from avoidable causes between 2001-2020 were calculated by region. Regional disparities in avoidable mortality were quantified on both absolute and relative scales. Trends and disparities in avoidable mortality were analyzed using joinpoint regression models.

**RESULTS:**

Avoidable, treatable, and preventable mortalities in Korea decreased at different rates over time by region. The largest decreases were in the non-Seoul-Capital non-metropolitan area for avoidable and preventable mortality rates and the non-Seoul-Capital metropolitan area for treatable mortality rates, despite the largest decline being in the Seoul Capital Area prior to around 2009. Absolute and relative regional disparities in avoidable and preventable mortalities generally decreased. Relative disparities in treatable mortality between areas widened. Regional disparities in all types of mortalities tended to improve after around 2009, especially among males. In females, disparities in avoidable, treatable, and preventable mortalities between areas improved less or even worsened.

**CONCLUSIONS:**

Trends and disparities in avoidable mortality across areas in Korea seem to have varied under the influence of diverse social changes. Enhancing health services to underserved areas and strengthening gender-oriented policies are needed to reduce regional disparities in avoidable mortality.

## INTRODUCTION

Avoidable mortality (AM) means premature death due to certain conditions that should not occur with timely and effective health care and public health interventions, and is used internationally as an indicator to measure and compare the performance of health systems [1]. AM has decreased in most European Union (EU) and Organization for Economic Co-operation and Development (OECD) countries [2,3]. Treatable mortality (TM) is on the decline worldwide, but the gap between countries has widened [4]. In South Korea (hereafter referred to as Korea), AM has decreased by almost 60% since 2000, reaching a level of 70% to 76% of the averages of the OECD and EU countries, respectively [2,3,5]. Korea has the largest reduction in TM and preventable mortality (PM) among high-income and OECD countries, making it one of the countries with the lowest TM and below-average PM [4-7].

Trends in AM can be influenced by demographic, socioeconomic, and political factors such as income, education, ethnicity, health expenditure, health system performance, and political regime type [4,6-9]. AM rates also differ across geographical areas at various levels [4,7,8], possibly due to unequal distribution of resources and health risks [8]. Korea has the second-largest share of population and gross domestic product (GDP) in metropolitan areas and the second-largest GDP gap between territorial level 2 regions among the OECD countries [10,11]. The Seoul Capital Area (SCA) refers to Seoul, the capital of Korea, and its surrounding provinces, occupying 12% of the country’s land area [12]. It is the sixth-largest metropolitan area in the world with 26 million inhabitants [13]. Most resources are concentrated in the SCA, accounting for 50% of the population, 53% of GDP, 55% of gross national income, and half or more of major medical resources (e.g., physicians, nurses, and tertiary hospitals) in 2020 [12]. While mental health is better in the non-SCA regions, health behaviors, injury prevention awareness, and non-communicable disease management are generally better in the SCA [14]. Accordingly, in Korea, reducing the concentration of resources in the mega-metropolitan SCA and the gap with the non-SCA regions has been an important policy agenda for over five decades, and mitigating health inequalities including regional disparities has been a key goal of the health system [15,16].

Metropolitan-area-related differences in AM were observed in several studies [17-21], some of which focused on disparities across metropolitan areas [17,18]. A few studies evaluated AM disparities between metropolitan and non-metropolitan areas; however, the results were inconsistent and research focusing on disparities between large metropolitan and non-metropolitan areas was scarce [19-21]. Previous findings in Korea showed that regional disparities in AM and PM have decreased, but those in TM have stagnated or increased [21]. However, these results were not suitable for international comparison due to underestimation of AM and did not present trend changes in AM rates and their regional disparities. Therefore, this study aimed to describe the regional AM trends in Korea and examine the trends in AM disparities between the SCA and non-SCA regions, thereby exploring the underlying reasons for the trend changes.

## MATERIALS AND METHODS

### Data

The number of deaths according to the International Classification of Diseases 10th Revision code from the death certificate registry [22] and mid-year population from resident registration statistics were obtained by sex, age, and administrative district from the data of Statistics Korea for 2001-2020 [12]. Mortality data before 2000 was not used, because of substantial underreporting of infant deaths [6,23]. Excluding 644 deaths of unknown age, 22,089 deaths outside Korea, and 2,655,153 deaths aged 75 years and over from 5,276,833 deaths during the data period, 2,598,968 all-cause deaths aged 0-74 years were extracted, of which 1,994,286 (76.7%) were avoidable deaths, composed of 557,549.5 treatable deaths and 1,436,736.5 preventable deaths.

### Variables

Age-standardized mortality rates (ASMRs) from avoidable, treatable, and preventable causes were calculated by sex and region as the number of deaths per 100,000 person-years aged 0-74 years for each cause, using the direct method based on the 2010 OECD Standard Population. Avoidable, treatable, and preventable deaths were defined according to the list jointly developed by the OECD/Eurostat [1] (Supplementary Matereial 1: Table S1).

Following the OECD regional typology [16], 17 administrative districts corresponding to the OECD territorial level 3 regions were categorized into the SCA (Seoul, Incheon, and Gyeonggi), the non-Seoul-Capital metropolitan area (NSC-MA) (Busan, Daegu, Gwangju, Daejeon, Ulsan, and Sejong), and the non-Seoul-Capital nonmetropolitan area (NSC-NMA) (Gangwon, Chungbuk, Chungnam, Jeonbuk, Jeonnam, Gyeongbuk, Gyeongnam, and Jeju). The population density per km2 was over 2,000 in the SCA and NSC-MA, and less than 200 in the NSC-NMA [12].

Because there is no consensus on the best strategy for measuring and interpreting health disparities due to the multidimensional complexity of the concept of inequality, an assessment of health disparities should comprehensively consider the broadest range of metrics [24]. Hence, disparities between the areas in ASMRs from avoidable causes were quantified on both absolute and relative scales: absolute disparity measures involved range difference (RD), between group variance (BGV), extended absolute concentration index, and slope index of inequality, and relative disparity measures involved range ratio (RR), index of disparity, mean log deviation, Theil index, extended relative concentration index, relative index of inequality, and Kunst-Mackenbach relative index [25]. The health disparity measures were calculated so that the higher the value, the higher the degree of disparity. Details of these measures are described in [25] Supplementary Material 2: Appendix A.

### Statistical analysis

Joinpoint regression analysis was employed to identify the points in time when the trends in AM rates and their regional disparities significantly changed and to estimate annual percent changes (APCs) with 95% confidence intervals (CIs) using the Monte Carlo permutation method [26] (Supplementary Material 2: Appendix B).

Analyses were performed using the Health Disparities Calculator (version 2.0.0) [25] and Joinpoint Regression Program (version 4.9.1.0) [26].

## RESULTS

**Figure 1.**
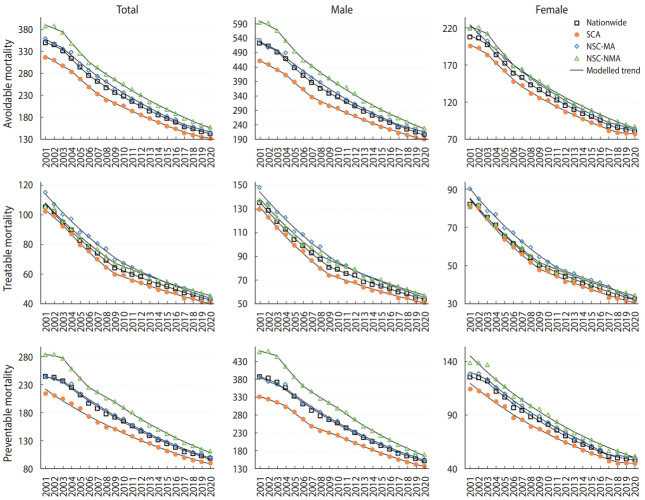
Trends in age-standardized mortality rates from avoidable, treatable, and preventable causes by sex and region in South Korea, 2001-2020. SCA, Seoul Capital Area; NSC-MA, non-Seoul-Capital metropolitan area; NSC-NMA, non-Seoul-Capital non-metropolitan area.

Trends and changes in ASMRs from avoidable, treatable, and preventable causes by sex and region in Korea from 2001 to 2020 are presented in Figure 1, Table 1, and Supplementary Material 3. AM decreased by 4.6% per year, explaining 87.4% of all-cause mortality reductions. TM and PM decreased to a similar extent, with the latter accounting for 69.9% of the decline in AM. Although ASMRs from all types of causes decreased most rapidly in the SCA until 2009, thereafter, AM and PM rates decreased fastest in the NSC-NMA and TM rates did so in the NSC-MA (Supplementary Material 1: Table S2).

The decreasing trends in AM accelerated from around 2004, with a larger decline in the NSC-NMA between 2003-2006 (APC, -6.4; 95% CI -8.6 to -4.2), after which it tended to slow down from around 2008, with a significant slope change for males. For females, the decreasing trend of AM tended to slow down from around 2018, mainly in the SCA and NSC-MA, which was also the case with PM in females. The TM rates decreased until around 2009 (APC, -6.1; 95% CI -6.3 to -5.9), and then the decline slowed down (APC, -3.7; 95% CI -3.9 to -3.6) for both sexes and in all three areas. With a faster decline in the NSC-MA than in the NSC-NMA after 2009, TM rates were highest in the NSC-NMA after around 2014. The PM rates remained unchanged until 2003 (APC, -2.1; 95% CI -4.5 to 0.4), then decreased from 2004 (APC, -5.0; 95% CI -5.2 to -4.8), with the fastest decline in the NSC-NMA (APC, -5.3; 95% CI -5.9 to -4.7) and the slowest decline in the SCA (APC, -4.7; 95% CI -4.8 to -4.5) between 2003- 2020. The slope change of PM from 2004 was significant in males (p = 0.047), but not in females (p = 0.064). Similar to AM, decreases in PM seemed to slow down from around 2008, mainly in the SCA and NSC-NMA among males, despite non-significant slope changes.

**Figure 2.**
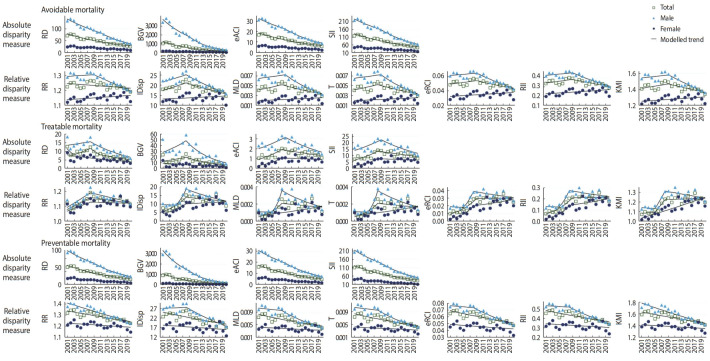
Trends in avoidable, treatable, and preventable mortality disparities between areas by sex and disparity measure in South Korea, 2001-2020. RD, range difference; BGV, between group variance; eACI, extended absolute concentration index; SII, slope index of inequality; RR, range ratio; IDisp, index of disparity; MLD, mean log deviation; T, Theil index; eRCI, extended relative concentration index; RII, relative index of inequality; KMI, Kunst-Mackenbach relative index.

Figure 2, Table 2, and Supplementary Material 3 show trends and changes in regional disparities in AM, TM, and PM. For all absolute and relative disparity measures and all types of mortalities, females had lower inequalities between areas but more unfavorable changes in regional inequalities than males. Absolute and relative disparities between areas in terms of PM were greater than for TM.

In males, absolute and relative disparities in AM decreased between 2001-2020. The decreasing trends of absolute disparities and the increasing or flat trends of relative disparities improved after around 2009. However, in females, absolute disparities decreased but relative disparities tended to increase throughout the study period. As for TM, inequalities tended to increase in all absolute and relative disparity measures save for RD and BGV. Absolute and relative regional disparities in TM, except for RD and BGV, increased until around 2009, and then declined or slowed down. Since around 2009, absolute and relative disparities decreased for males, but for females, disparities continued to increase in all relative measures, apart from RR, despite increasing trends slowing down. Regarding PM, absolute disparities decreased for both males and females, but relative disparities decreased only in males and did not decrease significantly in females excluding RR. In males, disparity reductions were greater than in females and the decline tended to accelerate after around 2009.

## DISCUSSION

Between 2001-2020, AM, TM, and PM in Korea decreased at different rates over time by region and sex. The largest decreases were in the NSC-NMA for AM and PM and the NSC-MA for TM, despite the largest decline being in the SCA before around 2009. Absolute and relative regional disparities in AM and PM tended to improve, especially in males since around 2009, but in females, they only narrowed in absolute disparities, with relative disparities not improving or even worsening. Relative disparities in TM between areas widened, particularly among females, despite improvement since around 2009.

Consistent with previous findings that AM decreased faster than all-cause mortality [4,6,7,9], nearly 90% of the decline in all-cause mortality between 2001-2020 was attributed to AM reduction, suggesting substantial improvements in health system in Korea [6]. The decrease in AM was led by PM reduction, but preventable deaths accounted for 70% of avoidable deaths, which was higher than approximately 64% in OECD and EU countries [2,5], indicating a need for decreasing PM.

The downward inflection of AM in 2004 reflected a concurrent decline in PM, which could have resulted from the implementation of the 2002 National Health Plan that included comprehensive health promotion programs to improve population health [6,15]. The Health Promotion Fund, heavily financed through two tobacco tax increases in 2002 and 2005, provided financial support for implementing the National Health Plan [6,15]. Although public health providers have only accounted for 10% of Korea’s health delivery system, which is dominated by the private sector with very limited public health functions, public health centers (PHCs) run by the government have played a crucial role in providing public health services and are mostly distributed in non-metropolitan areas [15,27]. Compared to the SCA and NSC-MA, between 2001-2007, the number of PHC personnel per population was three to four times higher and the number of personnel per PHC was 50% to 100% higher in the NSC-NMA [12], which might have contributed to a greater reduction in AM and PM from 2004 in the NSC-NMA [27,28]. The slowing tendency in AM and PM reductions during the 2008-2009 global financial crisis, although not as pronounced in PM, might be attributed to countercyclical changes in ASMRs from specific causes such as suicide and alcohol-related diseases [6,29]. The deceleration of the decreases in AM and PM after the crisis, compared to the pre-crisis trends, might suggest a countercyclical relationship with economic growth [30], which, in Korea, fell from an annual average of 5.2% between 2001-2007 to 2.9% between 2010-2020 [12]. The largest deceleration or the least acceleration in the downward trends of AM and PM in the SCA after the crisis might be because the SCA had a lower disposable income growth rate and a higher unemployment rate than the NSC-MA and NSC-NMA between 2010-2020 [12,30]. Most of the improvements in AM and PM in the NSC-NMA after 2009 might be ascribed to the advantage of human resources in PHCs and the highest disposable income gains [12,28].

TM appeared to not be affected during the crisis, similar to most EU countries [30,31], but to be inversely related to the share of current health expenditure (CHE) in GDP [9], whose annual growth rate averaged 4.6% for 2001-2009, slowed to 2.6% for 2010-2017, and then rose again to 5.8% for 2018-2020 [12]. The change in CHE per GDP almost coincided with the political regime change. Under the conservative governments (2008-2017), CHE increased slightly, which was led by an increase in private health expenditure, whereas under the liberal governments (2001-2008 and 2017-2020), CHE increased considerably, mainly due to an increase in public health expenditure [12]. TM that trends inversely accompanying CHE could be supported by the previous result on the association of lower TM with higher CHE and lower private health expenditure [9]. The non-significant acceleration of the decrease in TM from 2004 in the SCA, where large hospitals were concentrated, might be because the government’s mandatory external evaluation of the quality of medical care began in the mid-2000s at large hospitals with more potential to improve performance [15]. As the hospital performance evaluation was expanded to more medical institutions and clinical conditions and linked to pay-for-performance compensation, both hospitals’ performance and variations in quality between hospitals improved, especially among the lower performing hospitals [15,32], which might be a reason for the least deceleration of declining trends in TM after 2009 in the NSC-MA. Cardiovascular disease mortality, accounting for more than one-third of TM, was the highest in the NSC-MA, where the share of manufacturing industrial land use was the highest and the fraction of green space area was the lowest, increasing risks of cardiovascular death, which could have contributed to higher TM in the NSC-MA than in the NSC-NMA until the mid-2010s [33]. However, the NSC-MA had the largest number of major medical resources (e.g., tertiary hospitals, inpatient beds, specialist doctors, and high-tech medical equipment) per population and area, which might have minimized the adverse impact of post-crisis reversal and reduced increases in CHE [9,12,31,34], along with the effects of performance evaluation. These might also explain the accelerated tendency in decreasing trends of PM from 2015 and TM from 2018 in the NSC-MA, although evident only among females, possibly due to the expansion of health insurance coverage to reduce out-of-pocket payments in 2014 and 2017 [35]. Contrary to TM, decreases in AM and PM slowed from around 2018, which might be attributed to the increased instances of copycat suicides following celebrities’ suicides, especially in the SCA and NSC-MA among females, and a decrease in survival rates for cancers [12,36].

Regional PM disparities over time had moderate to strong positive correlations with the trend of income inequality across areas measured by the Gini coefficient of regional gross disposable income per capita (Supplementary Material 1: Table S3), which were consistent with the previous results that, within a country, mortality disparities changed with income inequality [37]. The Gini coefficient of regional income inequality fluctuated at around 0.035 between 2001-2008, and after a concave upward tendency during the crisis (0.029), reduced from 0.038 to 0.024 between 2011-2020 [12]. Notwithstanding the rise of labor income inequality due to deepening labor market polarization after the crisis, in Korea, disposable income inequality was reduced because of a decrease in real estate and financial income among the high-income group and an increase in transfer and social insurance income among the low-income group [38], narrowing the regional income inequality between the SCA with more high-income earners and the NSC-NMA with more low-income earners [39]. It could also explain the slowest decline in the SCA and the fastest decline in the NSC-NMA of PM after the crisis (Supplementary Material 1: Table S2).

Regional disparities in TM, which had little correlation with the regional income inequality trend [9] (Supplementary Material 1: Table S3), were smaller than in PM, which should be sensitive to income inequality when advantaged groups had more opportunities to prevent death [37]. Despite relatively equitable access to medical care by income under the single-payer universal health insurance [40], time costs incurred by the disproportionate distribution of medical resources could be barriers to medical use, possibly widening regional disparities in TM [34,41]. About 80% of tertiary hospitals and 78% of inpatient beds in general hospitals with over 500 beds were located in the SCA and NSC-MA, and the number of general hospital beds per area was the lowest in the NSC-NMA [12,42]. Therefore, the proportion of the population without timely access to tertiary or secondary care is negligible in the SCA and NSC-MA, whereas it far exceeded the national average in the NSC-NMA [43]. Unlike European countries [44], there was a negative association between mortality and the supply of beds in large hospitals in Korea, where mortality rates were lower for the population living in areas with over 500-bed hospitals than for those without [45]. Among acute beds, the share of beds in over 500-bed hospitals, the main targets for the government’s quality improvement programs, was the highest in the SCA, followed by the NSC-MA and NSC-NMA [12,45]. Accordingly, differences and changes in the timely access to quality medical care due to improved quality of care and reduced quality variations led by large hospitals in the SCA and NSC-MA might have made TM better in the NSC-MA compared to the NSC-NMA [32,44,45] and ameliorated growing regional disparities in TM.

Disparity trends in AM between areas resulted from the combined influences of regional disparity trends in PM and TM. Findings in European countries showed that relative socioeconomic inequalities in premature and amenable mortalities increased but absolute inequalities did not [9,46]. In Korea, absolute and relative disparities in AM and PM between areas generally decreased, regional disparity trends in TM changed similarly to those in Europe [9,46], and regional disparities in all types of mortalities tended to improve after around 2009, especially in males. However, in females, regional mortality disparities improved less or were even exacerbated, although the disparities were smaller in females, which did not necessarily indicate lower socioeconomic inequalities among females, given that there was no relationship between the magnitude of mortality disparities and the level of income inequalities [37]. Compared to males, Korean females earned one-third less wages and experienced the largest gender wage gap among OECD countries [12]. They were also less employed, worked mostly in precarious employment, had two times higher relative poverty rates, experienced more severe poverty for longer, and had a higher probability of entering and re-entering poverty and a lower probability of exiting poverty [12,47], making females less likely to benefit from socioeconomic changes and health policies. Furthermore, although the distribution of female-headed households did not differ by region [12], female householders in the NSC-NMA consistently had a much higher relative poverty rate and were older, less educated, unemployed, and more likely to live alone than those in metropolitan areas, and females in poverty were more prone to time poverty by spending time to earn income as well as on unpaid work, making it difficult to access quality health care in a timely manner [47-49], which might have resulted in less improvement or even the worsening of regional disparities in AM and TM among females.

This study has limitations. First, there have been concerns about the accuracy of cause-of-death data in Korea, such as underreporting or misreporting of deaths due to intentional injuries and the adverse effects of medical and surgical care, but the probability of misclassifying death causes would be low due to the relatively high fraction of deaths registered to well-certified causes (82.3% between 2000-2016) and the high proportion of physician-certified deaths [6,23,29,50]. Second, regional mortality trends and disparities across areas were not analyzed for each subgroup of causes of death, as it was beyond the scope of this study to explore the underlying reasons for changes in trends and disparities in AM. However, an analysis of mortality trends and disparities by subgroups of avoidable deaths will be needed in the future for a better understanding of the drivers of variation in AM. Third, the results from this study do not present any association or causality between AM and individual policies or social events but might help identify the plausible reasons for changes in AM. Finally, while what might have influenced the variations in AM was discussed based on available evidence, there may be other factors accounting for these variations. Research demonstrating factors that influenced variations in AM across regions has been scarce in Korea, suggesting the need for such studies.

Between 2001-2020, trends and disparities in AM, TM, and PM across areas in Korea seem to have varied under the influence of diverse social changes, including strengthening of public health policies, expansion of health insurance coverage, changes in CHE, national quality improvement programs, business cycle, income inequality changes, and feminization of poverty. Although the absolute and relative gaps in AM with the SCA have narrowed since around 2009, TM and PM are the highest in the NSC-NMA, implying the need for enhancing public health and health care services to underserved areas. Moreover, gender-oriented policies should be strengthened to close the less improved or widening gaps in AM and TM among females. Further studies are needed to elucidate changes in regional disparities in AM.

## Figures and Tables

**Table 1.** Changes in age-standardized mortality rates from avoidable, treatable, and preventable causes by gender and region in Korea, 2001-2020

Mortality	Sex	Nationwide			Seoul Capital area			Non-Seoul-Capital metropolitan area			Non-Seoul-Capital non-metropolitan area		
		Period	APC (95% CI)	p-value for slope change	Period	APC (95% CI)	p-value for slope change	Period	APC (95% CI)	p-value for slope change	Period	APC (95% CI)	p-value for slope change
Avoidable mortality	Total	2001-2003	-2.5 (-4.6, -0.3)	-	2001-2004	-3.6 (-5.2, -2.0)	-	2001-2003	-3.0 (-6.5, 0.7)	-	2001-2003	-2.2 (-4.3, 0.1)	-
		2003-2006	-6.2 (-8.3, -4.1)	0.020	2004-2007	-6.5 (-9.6, -3.3)	0.101	2003-2017	-5.0 (-5.1, -4.9)	0.242	2003-2006	-6.4 (-8.6, -4.2)	0.012
		2006-2018	-4.8 (-4.9, -4.6)	0.158	2007-2018	-4.5 (-4.8, -4.3)	0.196				2006-2020	-4.7 (-4.9, -4.6)	0.129
		2018-2020	-3.3 (-5.8, -0.8)	0.215	2018-2020	-2.9 (-6.4, 0.6)	0.328	2017-2020	-3.6 (-5.2, -2.0)	0.088			
		2001-2020	-4.6 (-5.0, -4.2)		2001-2020	-4.5 (-5.1, -3.9)		2001-2020	-4.6 (-5.0, -4.2)		2001-2020	-4.7 (-5.1, -4.3)	
	Male	2001-2003	-2.8 (-5.1, -0.5)	-	2001-2004	-3.6 (-4.9, -2.2)	-	2001-2003	-2.8 (-7.4, 2.1)	-	2001-2003	-2.6 (-5.3, 0.1)	-
		2003-2007	-6.0 (-7.1, -4.9)	0.018	2004-2007	-6.7 (-9.2, -4.2)	0.033	2003-2017	-5.1 (-5.2, -4.9)	0.314	2003-2006	-6.3 (-8.9, -3.5)	0.057
		2007-2020	-4.8 (-4.9, -4.7)	0.038	2007-2020	-4.4 (-4.5, -4.2)	0.063				2006-2020	-5.0 (-5.2, -4.9)	0.344
								2017-2020	-3.7 (-5.5, -1.8)	0.122			
		2001-2020	-4.8 (-5.1, -4.5)		2001-2020	-4.6 (-5.0, -4.2)		2001-2020	-4.6 (-5.1, -4.1)		2001-2020	-5.0 (-5.4, -4.5)	
	Female	2001-2003	-2.8 (-6.4, 0.9)	-	2001-2003	-3.2 (-7.8, 1.7)	-	2001-2017	-5.4 (-5.5, -5.3)	-	2001-2003	-2.1 (-6.1, 2.0)	-
		2003-2006	-6.9 (-10.2, -3.5)	0.096	2003-2007	-6.7 (-8.4, -4.9)	0.151				2003-2006	-7.2 (-11.1, -3.0)	0.078
		2006-2018	-5.1 (-5.3, -5.0)	0.271	2007-2018	-5.1 (-5.3, -4.9)	0.079				2006-2020	-4.9 (-5.1, -4.7)	0.243
		2018-2020	-1.9 (-6.5, 2.9)	0.150	2018-2020	-1.1 (-6.5, 4.6)	0.133	2017-2020	-3.4 (-5.3, -1.4)	0.046			
		2001-2020	-4.8 (-5.6, -4.1)		2001-2020	-4.8 (-5.6, -4.1)		2001-2020	-5.1 (-5.4, -4.8)		2001-2020	-4.9 (-5.7, -4.2)	
Treatable mortality	Total	2001-2009	-6.1 (-6.3, -5.9)	-	2001-2003	-5.1 (-9.5, -0.4)	-	2001-2010	-5.7 (-6.0, -5.4)	-	2001-2009	-5.7 (-6.0, -5.3)	-
					2003-2009	-6.9 (-7.5, -6.4)	0.389						
		2009-2020	-3.7 (-3.9, -3.6)	<0.001	2009-2020	-3.6 (-3.8, -3.5)	<0.001	2010-2020	-4.2 (-4.4, -3.9)	<0.001	2009-2020	-3.5 (-3.7, -3.2)	<0.001
		2001-2020	-4.7 (-4.9, -4.6)		2001-2020	-4.8 (-5.3, -4.4)		2001-2020	-4.9 (-5.1, -4.7)		2001-2020	-4.4 (-4.6, -4.2)	
	Male	2001-2009	-6.2 (-6.4, -5.9)	-	2001-2009	-6.9 (-7.2, -6.6)	-	2001-2010	-5.7 (-6.1, -5.4)	-	2001-2008	-5.9 (-6.4, -5.4)	-
		2009-2020	-3.7 (-3.8, -3.5)	<0.001	2009-2020	-3.5 (-3.7, -3.3)	<0.001	2010-2020	-4.1 (-4.5, -3.8)	<0.001	2008-2020	-3.7 (-4.0, -3.5)	<0.001
		2001-2020	-4.7 (-4.9, -4.6)		2001-2020	-4.9 (-5.1, -4.8)		2001-2020	-4.9 (-5.1, -4.7)		2001-2020	-4.5 (-4.8, -4.3)	
	Female	2001-2009	-6.3 (-6.6, -5.9)	-	2001-2009	-6.8 (-7.2, -6.3)	-	2001-2011	-5.9 (-6.1, -5.7)	-	2001-2009	-5.9 (-6.4, -5.5)	-
		2009-2020	-4.0 (-4.3, -3.8)	<0.001	2009-2020	-4.1 (-4.4, -3.8)	<0.001	2011-2017	-3.7 (-4.5, -3.0)	<0.001	2009-2020	-3.7 (-4.0, -3.4)	<0.001
								2017-2020	-6.2 (-8.2, -4.0)	0.036			
		2001-2020	-5.0 (-5.2, -4.8)		2001-2020	-5.2 (-5.5, -5.0)		2001-2020	-5.3 (-5.6, -4.9)		2001-2020	-4.6 (-4.9, -4.4)	
Preventable mortality	Total	2001-2003	-2.1 (-4.5, 0.4)	-	2001-2020	-4.7 (-4.8, -4.5)	-	2001-2003	-1.3 (-6.6, 4.3)	-	2001-2003	-1.1 (-3.6, 1.4)	-
		2003-2020	-5.0 (-5.2, -4.8)	0.021				2003-2020	-5.0 (-5.1, -4.8)	0.167	2003-2006	-6.7 (-9.1, -4.3)	0.005
											2006-2009	-4.0 (-6.6, -1.4)	0.117
											2009-2020	-5.3 (-5.5, -5.0)	0.304
		2001-2020	-4.7 (-5.0, -4.4)		2001-2020	-4.7 (-4.8, -4.5)		2001-2020	-4.6 (-5.1, -4.1)		2001-2020	-4.9 (-5.4, -4.3)	
	Male	2001-2003	-2.5 (-5.1, 0.2)	-	2001-2004	-2.7 (-4.1, -1.3)	-	2001-2004	-2.7 (-4.9, -0.6)	-	2001-2003	-1.5 (-4.5, 1.5)	-
		2003-2020	-5.1 (-5.3, -5.0)	0.047	2004-2007	-6.6 (-9.0, -4.3)	0.010	2004-2020	-5.0 (-5.2, -4.9)	0.035	2003-2006	-6.7 (-9.6, -3.8)	0.020
					2007-2010	-3.6 (-5.9, -1.2)	0.068				2006-2010	-4.6 (-6.1, -3.0)	0.174
					2010-2020	-4.8 (-5.0, -4.5)	0.290				2010-2020	-5.7 (-5.9, -5.4)	0.139
		2001-2020	-4.9 (-5.1, -4.6)		2001-2020	-4.6 (-5.1, -4.0)		2001-2020	-4.7 (-5.0, -4.4)		2001-2020	-5.2 (-5.7, -4.6)	
	Female	2001-2003	-2.3 (-5.9, 1.3)	-	2001-2018	-5.4 (-5.6, -5.2)	-	2001-2003	-2.7 (-7.0, 1.7)	-	2001-2020	-5.4 (-5.6, -5.1)	-
		2003-2017	-5.7 (-5.9, -5.4)	0.064				2003-2014	-5.5 (-5.8, -5.3)	0.178			
								2014-2017	-6.7 (-11.4, -1.8)	0.586			
		2017-2020	-3.0 (-5.4, -0.5)	0.034	2018-2020	-1.1 (-8.2, 6.6)	0.225	2017-2020	-1.6 (-4.0, 0.7)	0.063			
		2001-2020	-4.9 (-5.4, -4.4)		2001-2020	-5.0 (-5.7, -4.2)		2001-2020	-4.8 (-5.7, -4.0)		2001-2020	-5.4 (-5.6, -5.1)	

APC, annual percent change; CI, confidence interval.

**Table 2.** Changes in avoidable, treatable, and preventable mortality disparities between areas by gender and disparity measure in Korea, 2001-2020

Mortality	Disparity measure		Total			Male			Female		
			Period	APC (95% CI)	p-value for slope change	Period	APC (95% CI)	p-value for slope change	Period	APC (95% CI)	p-value for slope change
Avoidable mortality	Absolute disparity	RD	2001-2020	-5.4 (-6.1, -4.8)	-	2001-2011	-5.1 (-6.3, -4.0)	-	2001-2020	-4.3 (-5.1, -3.4)	-
						2011-2020	-8.6 (-10.4, -6.7)	0.004			
			2001-2020	-5.4 (-6.1, -4.8)		2001-2020	-6.8 (-7.8, -5.8)		2001-2020	-4.3 (-5.1, -3.4)	
		BGV	2001-2020	-10.6 (-11.7, -9.5)	-	2001-2009	-8.9 (-12.0, -5.6)	-	2001-2020	-8.9 (-10.4, -7.3)	-
						2009-2020	-15.4 (-17.8, -12.9)	0.004			
			2001-2020	-10.6 (-11.7, -9.5)		2001-2020	-12.7 (-14.5, -10.9)		2001-2020	-8.9 (-10.4, -7.3)	
		eACI	2001-2020	-5.5 (-6.1, -4.9)	-	2001-2009	-4.6 (-6.2, -2.9)	-	2001-2020	-4.4 (-5.3, -3.6)	-
						2009-2020	-8.0 (-9.3, -6.7)	0.003			
			2001-2020	-5.5 (-6.1, -4.9)		2001-2020	-6.6 (-7.5, -5.6)		2001-2020	-4.4 (-5.3, -3.6)	
		SII	2001-2020	-5.4 (-6.0, -4.8)	-	2001-2009	-4.4 (-6.0, -2.8)	-	2001-2020	-4.3 (-5.1, -3.5)	-
						2009-2020	-8.0 (-9.3, -6.7)	0.002			
			2001-2020	-5.4 (-6.0, -4.8)		2001-2020	-6.5 (-7.4, -5.6)		2001-2020	-4.3 (-5.1, -3.5)	
	Relative disparity	RR	2001-2020	-0.1 (-0.3, 0.0)	-	2001-2009	0.1 (-0.3, 0.5)	-	2001-2020	0.1 (0.0, 0.3)	-
						2009-2020	-0.8 (-1.1, -0.5)	0.002			
			2001-2020	-0.1 (-0.3, 0.0)		2001-2020	-0.4 (-0.6, -0.2)		2001-2020	0.1 (0.0, 0.3)	
		IDisp	2001-2008	2.7 (0.5, 5.0)	-	2001-2008	2.2 (0.2, 4.3)	-	2001-2020	0.6 (-0.4, 1.6)	-
			2008-2020	-2.4 (-3.5, -1.4)	<0.001	2008-2020	-3.6 (-4.6, -2.6)	<0.001			
			2001-2020	-0.6 (-1.5, 0.4)		2001-2020	-1.5 (-2.4, -0.6)		2001-2020	0.6 (-0.4, 1.6)	
		MLD	2001-2008	3.3 (-1.1, 7.9)	-	2001-2009	1.4 (-1.9, 4.7)	-	2001-2020	1.4 (-0.4, 3.3)	-
			2008-2020	-3.7 (-5.9, -1.6)	0.007	2009-2020	-6.7 (-9.1, -4.3)	<0.001			
			2001-2020	-1.2 (-3.1, 0.8)		2001-2020	-3.4 (-5.2, -1.6)		2001-2020	1.4 (-0.4, 3.3)	
		T	2001-2008	3.3 (-1.1, 7.9)	-	2001-2009	1.3 (-1.9, 4.7)	-	2001-2020	1.4 (-0.4, 3.3)	-
			2008-2020	-3.7 (-5.8, -1.5)	0.008	2009-2020	-6.7 (-9.1, -4.2)	<0.001			
			2001-2020	-1.2 (-3.1, 0.8)		2001-2020	-3.4 (-5.2, -1.6)		2001-2020	1.4 (-0.4, 3.3)	
		eRCI	2001-2009	1.2 (-0.7, 3.0)	-	2001-2009	0.6 (-1.0, 2.2)	-	2001-2020	0.8 (-0.1, 1.8)	-
			2009-2020	-2.0 (-3.4, -0.7)	0.009	2009-2020	-3.4 (-4.7, -2.2)	<0.001			
			2001-2020	-0.7 (-1.7, 0.3)		2001-2020	-1.7 (-2.7, -0.8)		2001-2020	0.8 (-0.1, 1.8)	
		RII	2001-2009	1.3 (-0.5, 3.2)	-	2001-2009	0.8 (-0.8, 2.4)	-	2001-2020	1.0 (0.0, 1.9)	-
			2009-2020	-2.0 (-3.3, -0.6)	0.007	2009-2020	-3.4 (-4.6, -2.1)	<0.001			
			2001-2020	-0.6 (-1.6, 0.4)		2001-2020	-1.7 (-2.6, -0.7)		2001-2020	1.0 (0.0, 1.9)	
		KMI	2001-2009	0.5 (-0.2, 1.2)	-	2001-2009	0.3 (-0.5, 1.1)	-	2001-2020	0.2 (0.0, 0.5)	-
			2009-2020	-0.7 (-1.2, -0.2)	0.011	2009-2020	-1.3 (-1.8, -0.9)	0.001			
			2001-2020	-0.2 (-0.6, 0.2)		2001-2020	-0.7 (-1.0, -0.3)		2001-2020	0.2 (0.0, 0.5)	
Treatable mortality	Absolute disparity	RD	2001-2003	-16.1 (-46.8, 32.5)	-	2001-2008	2.1 (-2.2, 6.5)	-	2001-2020	-3.6 (-4.8, -2.3)	-
			2003-2008	7.8 (0.3, 15.9)	0.251						
			2008-2011	-12.1 (-34.4, 17.8)	0.161	2008-2020	-7.0 (-8.4, -5.5)	0.004			
			2011-2020	-4.6 (-6.2, -3.0)	0.544						
			2001-2020	-4.0 (-9.7, 1.9)		2001-2020	-3.7 (-5.4, -2.1)		2001-2020	-3.6 (-4.8, -2.3)	
		BGV	2001-2008	6.6 (-3.3, 17.5)	-	2001-2008	8.0 (-1.8, 18.9)	-	2001-2020	-5.2 (-7.7, -2.6)	-
			2008-2020	-9.6 (-12.5, -6.5)	0.004	2008-2020	-12.3 (-15.2, -9.3)	<0.001			
			2001-2020	-3.9 (-7.5, -0.2)		2001-2020	-5.3 (-8.8, -1.6)		2001-2020	-5.2 (-7.7, -2.6)	
		eACI	2001-2008	10.8 (4.4, 17.6)		2001-2009	5.8 (1.1, 10.8)		2001-2008	19.5 (4.3, 37.1)	
			2008-2020	-3.2 (-4.6, -1.8)	<0.001	2009-2020	-5.7 (-7.7, -3.7)	<0.001	2008-2020	-1.4 (-3.5, 0.7)	0.010
			2001-2020	1.7 (-0.4, 4.0)		2001-2020	-1.0 (-3.1, 1.1)		2001-2020	5.9 (0.9, 11.1)	
		SII	2001-2008	11.0 (4.7, 17.8)	-	2001-2009	6.0 (1.3, 10.9)	-	2001-2008	19.8 (4.5, 37.3)	-
			2008-2020	-3.1 (-4.5, -1.8)	<0.001	2009-2020	-5.7 (-7.7, -3.7)	<0.001	2008-2020	-1.3 (-3.4, 0.8)	0.009
			2001-2020	1.9 (-0.3, 4.1)		2001-2020	-0.9 (-3.0, 1.2)		2001-2020	6.0 (1.0, 11.2)	
	Relative disparity	RR	2001-2008	1.0 (0.6, 1.5)	-	2001-2008	1.2 (0.7, 1.8)	-	2001-2008	0.8 (0.1, 1.4)	-
			2008-2020	-0.3 (-0.4, -0.1)	<0.001	2008-2020	-0.4 (-0.7, -0.2)	<0.001	2008-2020	-0.1 (-0.3, 0.2)	0.021
			2001-2020	0.2 (0.0, 0.4)		2001-2020	0.2 (0.0, 0.4)		2001-2020	0.3 (0.0, 0.5)	
		IDisp	2001-2008	13.9 (7.6, 20.7)	-	2001-2005	-0.1 (-12.5, 14.1)	-	2001-2008	12.7 (3.9, 22.2)	-
						2005-2008	25.3 (-19.2, 94.3)	0.303			
			2008-2020	-1.5 (-3.3, 0.4)	<0.001	2008-2020	-3.8 (-5.3, -2.2)	0.215	2008-2020	1.0 (-2.0, 4.0)	0.016
			2001-2020	4.0 (1.7, 6.3)		2001-2020	1.1 (-5.5, 8.2)		2001-2020	5.1 (1.8, 8.6)	
		MLD	2001-2008	22.4 (11.4, 34.6)	-	2001-2005	-0.5 (-22.2, 27.3)	-	2001-2020	4.9 (1.8, 8.1)	-
						2005-2008	49.2 (-33.7, 235.4)	0.318			
			2008-2020	-2.0 (-5.1, 1.2)	<0.001	2008-2020	-6.1 (-8.9, -3.3)	0.237			
			2001-2020	6.4 (2.5, 10.4)		2001-2020	2.2 (-9.8, 15.9)		2001-2020	4.9 (1.8, 8.1)	
		T	2001-2008	22.3 (11.2, 34.5)	-	2001-2005	-0.4 (-22.2, 27.6)	-	2001-2020	4.8 (1.7, 8.0)	-
						2005-2008	48.8 (-34.0, 235.4)	0.323			
			2008-2020	-2.0 (-5.1, 1.2)	<0.001	2008-2020	-6.1 (-8.9, -3.3)	0.241			
			2001-2020	6.3 (2.4, 10.3)		2001-2020	2.2 (-9.8, 15.9)		2001-2020	4.8 (1.7, 8.0)	
		eRCI	2001-2009	15.8 (10.5, 21.4)	-	2001-2005	-0.9 (-15.9, 16.8)	-	2001-2008	28.6 (11.7, 47.9)	-
						2005-2008	28.9 (-24.5, 120.1)	0.327			
			2009-2020	0.1 (-1.7, 1.9)	<0.001	2008-2020	-1.9 (-3.3, -0.5)	0.288	2008-2020	2.9 (0.7, 5.2)	0.004
			2001-2020	6.4 (4.3, 8.6)		2001-2020	2.6 (-5.5, 11.5)		2001-2020	11.7 (6.3, 17.3)	
		RII	2001-2009	16.0 (10.7, 21.6)		2001-2005	-0.7 (-15.7, 17.0)		2001-2008	28.8 (12.0, 48.2)	
						2005-2008	29.1 (-24.3, 120.1)	0.326			
			2009-2020	0.1 (-1.6, 1.9)	<0.001	2008-2020	-1.9 (-3.2, -0.4)	0.285	2008-2020	3.0 (0.7, 5.3)	0.004
			2001-2020	6.5 (4.4, 8.7)		2001-2020	2.7 (-5.4, 11.6)		2001-2020	11.8 (6.5, 17.5)	
		KMI	2001-2005	0.3 (-1.4, 2.0)	-	2001-2005	-0.3 (-2.3, 1.7)	-	2001-2020	1.0 (0.8, 1.2)	-
			2005-2008	3.9 (-3.7, 12.1)	0.341	2005-2008	5.2 (-3.5, 14.7)	0.215			
			2008-2020	0.1 (-0.2, 0.4)	0.307	2008-2020	-0.5 (-0.8, -0.1)	0.190			
			2001-2020	0.7 (-0.4, 1.9)		2001-2020	0.4 (-0.9, 1.8)		2001-2020	1.0 (0.8, 1.2)	
Preventable mortality	Absolute disparity	RD	2001-2020	-6.3 (-6.9, -5.7)	-	2001-2009	-5.6 (-7.1, -4.2)	-	2001-2020	-6.0 (-6.9, -5.2)	-
						2009-2020	-8.6 (-9.8, -7.3)	0.006			
			2001-2020	-6.3 (-6.9, -5.7)		2001-2020	-7.3 (-8.2, -6.4)		2001-2020	-6.0 (-6.9, -5.2)	
		BGV	2001-2020	-12.2 (-13.4, -11.0)	-	2001-2009	-11.0 (-13.7, -8.1)	-	2001-2020	-11.7 (-13.2, -10.2)	-
						2009-2020	-16.2 (-18.5, -13.8)	0.008			
			2001-2020	-12.2 (-13.4, -11.0)		2001-2020	-14.0 (-15.7, -12.3)		2001-2020	-11.7 (-13.2, -10.2)	
		eACI	2001-2020	-6.3 (-7.0, -5.7)	-	2001-2009	-5.6 (-7.0, -4.2)	-	2001-2020	-6.1 (-6.9, -5.3)	-
						2009-2020	-8.5 (-9.7, -7.3)	0.005			
			2001-2020	-6.3 (-7.0, -5.7)		2001-2020	-7.3 (-8.1, -6.4)		2001-2020	-6.1 (-6.9, -5.3)	
		SII	2001-2020	-6.2 (-6.9, -5.6)	-	2001-2009	-5.5 (-6.9, -4.0)	-	2001-2020	-6.0 (-6.8, -5.2)	-
						2009-2020	-8.5 (-9.7, -7.2)	0.004			
			2001-2020	-6.2 (-6.9, -5.6)		2001-2020	-7.2 (-8.1, -6.3)		2001-2020	-6.0 (-6.8, -5.2)	
	Relative disparity	RR	2001-2020	-0.4 (-0.5, -0.3)		2001-2020	-0.7 (-0.8, -0.5)		2001-2020	-0.2 (-0.3, -0.0)	
		IDisp	2001-2020	-1.5 (-2.1, -0.9)	-	2001-2008	0.3 (-1.7, 2.3)	-	2001-2020	-0.7 (-1.6, 0.2)	-
						2008-2020	-3.6 (-4.6, -2.6)	0.002			
			2001-2020	-1.5 (-2.1, -0.9)		2001-2020	-2.2 (-3.1, -1.3)		2001-2020	-0.7 (-1.6, 0.2)	
		MLD	2001-2020	-3.1 (-4.1, -2.0)	-	2001-2008	-1.1 (-4.4, 2.2)	-	2001-2020	-1.4 (-3.1, 0.3)	-
						2008-2020	-6.6 (-8.5, -4.6)	0.008			
			2001-2020	-3.1 (-4.1, -2.0)		2001-2020	-4.6 (-6.2, -3.0)		2001-2020	-1.4 (-3.1, 0.3)	
		T	2001-2020	-3.1 (-4.1, -2.0)	-	2001-2008	-1.2 (-4.5, 2.2)	-	2001-2020	-1.4 (-3.0, 0.3)	-
						2008-2020	-6.6 (-8.5, -4.6)	0.009			
			2001-2020	-3.1 (-4.1, -2.0)		2001-2020	-4.6 (-6.2, -3.0)		2001-2020	-1.4 (-3.0, 0.3)	
		eRCI	2001-2020	-1.6 (-2.1, -1.1)	-	2001-2008	-0.6 (-2.2, 1.1)	-	2001-2020	-0.8 (-1.6, 0.1)	-
						2008-2020	-3.4 (-4.4, -2.4)	0.006			
			2001-2020	-1.6 (-2.1, -1.1)		2001-2020	-2.4 (-3.2, -1.6)		2001-2020	-0.8 (-1.6, 0.1)	
		RII	2001-2020	-1.5 (-2.0, -0.9)	-	2001-2008	-0.4 (-2.0, 1.3)	-	2001-2020	-0.6 (-1.5, 0.2)	-
						2008-2020	-3.4 (-4.3, -2.4)	0.005			
			2001-2020	-1.5 (-2.0, -0.9)		2001-2020	-2.3 (-3.1, -1.5)		2001-2020	-0.6 (-1.5, 0.2)	
		KMI	2001-2020	-0.7 (-0.9, -0.4)		2001-2020	-1.1 (-1.4, -0.9)		2001-2020	-0.2 (-0.5, 0.0)	

APC, annual percent change; CI, confidence interval; RD, range difference; BGV, between group variance; eACI, extended absolute concentration index; SII, slope index of inequality; RR, range ratio; IDisp, index of disparity; MLD, mean log deviation; T, Theil index; eRCI, extended relative concentration index; RII, relative index of inequality; KMI, Kunst-Mackenbach relative index.

## SUPPLEMENTARY MATERIALS

Supplementary Material 1. Table S1. Definition of avoidable, treatable, and preventable deaths jointly developed by the OECD/Eurostat [1] Table S2. Changes in age-standardized mortality rates from avoidable, treatable, and preventable causes before and after 2009 by sex and region in South Korea Table S3. Correlation coefficients between the Gini coefficients of regional gross disposa-ble income per capita and disparities in avoidable, treatable, and preventable mortalities across areas in South Korea, 2001-2020

Supplementary Material 2. Appendix A: definition of health disparity measures [1-3] Appendix B: joinpoint regression model [4-8]

## References

[1] Organization for Economic Cooperation and Development (OECD) (2022). Avoidable mortality: OECD/Eurostat lists of preventable and treatable causes of death (January 2022 version). https://www.oecd.org/health/health-systems/Avoidable-mortality-2019-Joint-OECD-Eurostat-List-preventable-treatable-causes-of-death.pdf.

[2] Eurostat (2021). Treatable and preventable mortality of residents by cause and sex. https://ec.europa.eu/eurostat/databrowser/view/hlth_cd_apr/default/table?lang=en.

[3] Organization for Economic Cooperation and Development (OECD) (2021). Health status: avoidable mortality. https://stats.oecd.org/index.aspx?queryid=96018.

[4] GBD 2015 Healthcare Access and Quality Collaborators (2017). Healthcare Access and Quality Index based on mortality from causes amenable to personal health care in 195 countries and territories, 1990-2015: a novel analysis from the Global Burden of Disease Study 2015. Lancet.

[5] Organization for Economic Cooperation and Development (OECD) (2021). Health at a glance 2021: OECD indicators. https://www.oecd.org/health/health-at-a-glance/.

[6] Eun SJ (2019). Avoidable, amenable, and preventable mortalities in South Korea, 2000-2017: age-period-cohort trends and impact on life expectancy at birth. Soc Sci Med.

[7] Gianino MM, Lenzi J, Muça A, Fantini MP, Siliquini R, Ricciardi W (2017). Declining amenable mortality: time trend (2000-2013) and geographic area analysis. Health Serv Res.

[8] Subedi R, Greenberg TL, Roshanafshar S (2019). Does geography matter in mortality? An analysis of potentially avoidable mortality by remoteness index in Canada. Health Rep.

[9] Mackenbach JP, Hu Y, Artnik B, Bopp M, Costa G, Kalediene R (2017). Trends in inequalities in mortality amenable to health care in 17 European countries. Health Aff (Millwood).

[10] Organization for Economic Cooperation and Development (OECD) (2020). OECD regions and cities at a glance 2020. https://www.oecd.org/cfe/oecd-regions-andcities-at-a-glance-26173212.htm.

[11] Kim T, Kim E, Shin HS, Park MR, Lee HJ (2021). Comparison of income and employment disparities between regions in Korea and OECD countries. https://www.krihs.re.kr/board.es?mid=a10607000000&bid=0008&tag=&act=view&list_no=346058.

[12] Statistics Korea (2022). Korean Statistical Information Service. https://kosis.kr/eng/.

[13] Organization for Economic Cooperation and Development (OECD) (2020). Cities in the world: a new perspective on urbanisation. https://www.oecd.org/publications/cities-in-the-world-d0efcbda-en.htm.

[14] Korea Disease Control and Prevention Agency (2021). Korea community health at a glance 2020: Korea Community Health Survey (KCHS). https://chs.kdca.go.kr/chs/stats/statsMain.do.

[15] World Health Organization (2015). Republic of Korea health system review. https://apps.who.int/iris/handle/10665/208215.

[16] Organization for Economic Cooperation and Development (OECD) (2021). Perspectives on decentralisation and rural-urban linkages in Korea. https://www.oecd.org/publications/perspectives-on-decentralisation-and-rural-urban-linkages-in-korea-a3c685a7-en.htm.

[17] Weisz D, Gusmano MK, Rodwin VG, Neuberg LG (2008). Population health and the health system: a comparative analysis of avoidable mortality in three nations and their world cities. Eur J Public Health.

[18] Costa C, Santana P, Dimitroulopoulou S, Burstrom B, Borrell C, Schweikart J (2019). Population health inequalities across and within European metropolitan areas through the lens of the EURO-HEALTHY Population Health Index. Int J Environ Res Public Health.

[19] Lehikoinen M, Arffman M, Manderbacka K, Elovainio M, Keskimäki I (2016). Comparative observational study of mortality amenable by health policy and care between rural and urban Finland: no excess segregation of mortality in the capital despite its increasing residential differentiation. Int J Equity Health.

[20] Mühlichen M (2018). Avoidable mortality in the German Baltic Sea region since reunification: convergence or persistent disparities?. Eur J Popul.

[21] Choi MH, Moon MH, Yoon TH (2022). Avoidable mortality between metropolitan and non-metropolitan areas in Korea from 1995 to 2019: a descriptive study of implications for the national healthcare policy. Int J Environ Res Public Health.

[22] (2022). Microdata Integrated Service.

[23] Jo MW, Khang YH, Yun S, Lee JY, Lee MS, Lee SI (2004). Proportion of death certificates issued by physicians and associated factors in Korea, 1990-2002. J Prev Med Public Health.

[24] Harper S, King NB, Meersman SC, Reichman ME, Breen N, Lynch J (2010). Implicit value judgments in the measurement of health inequalities. Milbank Q.

[25] National Cancer Institute https://seer.cancer.gov/hdcalc/.

[26] National Cancer Institute https://surveillance.cancer.gov/joinpoint/.

[27] Organization for Economic Cooperation and Development (OECD) (2020). OECD reviews of public health: Korea: a healthier tomorrow. https://www.oecd.org/health/oecd-reviews-of-public-health-korea-be2b7063-en.htm.

[28] Do YK, Park K (2009). Local governments’ dependence on tobacco tax revenue: a deterrent to tobacco control in the Republic of Korea. Bull World Health Organ.

[29] Khang YH, Lynch JW, Kaplan GA (2005). Impact of economic crisis on cause-specific mortality in South Korea. Int J Epidemiol.

[30] Catalano R, Goldman-Mellor S, Saxton K, Margerison-Zilko C, Subbaraman M, LeWinn K (2011). The health effects of economic decline. Annu Rev Public Health.

[31] Karanikolos M, Mackenbach JP, Nolte E, Stuckler D, McKee M (2018). Amenable mortality in the EU-has the crisis changed its course?. Eur J Public Health.

[32] Kim SM, Jang WM, Ahn HA, Park HJ, Ahn HS (2012). Korean National Health Insurance value incentive program: achievements and future directions. J Prev Med Public Health.

[33] Kang HJ, Kwon S (2016). Regional disparity of cardiovascular mortality and its determinants. Health Policy Manag.

[34] Chang I, Kim BH (2019). Regional disparity of medical resources and its effect on age-standardized mortality rates in Korea. Ann Reg Sci.

[35] Yeo N (2020). Policy on non-reimbursable services in the National Health Insurance. Health Welf Policy Forum.

[36] National Cancer Center (2021). Cancer trends report 2021. https://www.cancerdata.kr/surveillance/boardView/referenceRoom?postSeq=71.

[37] Truesdale BC, Jencks C (2016). The Health effects of income inequality: averages and disparities. Annu Rev Public Health.

[38] Choi J, Kim S, Park S (2018). Income inequality in Korea in the post global financial crisis period. Korean J Econ Stud.

[39] Lee SH (2019). Regional job quality and socioeconomic inequality. https://keis.or.kr/user/extra/main/2405/publication/reportList/jsp/LayOutPage.do?categoryIdx=129&pubIdx=5187&reportIdx=4923&spage=14.

[40] Lu JF, Leung GM, Kwon S, Tin KY, Van Doorslaer E, O’Donnell O (2007). Horizontal equity in health care utilization evidence from three high-income Asian economies. Soc Sci Med.

[41] Kim S, Kwon S (2014). Has the National Health Insurance improved the inequality in the use of tertiary-care hospitals in Korea?. Health Policy.

[42] Ministry of Health and Welfare Health and welfare statistical yearbook 2002-2021 [cited 2022 May 17].

[43] National Medical Center (2020). Statistics on public healthcare in 2019. https://www.nmc.or.kr/nmc/bbs/B0000058/view.do?nttId=586&menuNo=200326&pageIn.

[44] Nolte E, McKee M (2004). Does healthcare save lives? Avoidable mortality revisited. https://www.nuffieldtrust.org.uk/files/2017-01/does-healthcare-savelives-web-final.pdf.

[45] Kim Y, Lee T, Park SK, Lee HY, Hwang SS, Kwak MY (2018). Korean National Health Insurance Atlas (KNHI-Atlas) project, third study.

[46] Mackenbach JP, Kulhánová I, Artnik B, Bopp M, Borrell C, Clemens T (2016). Changes in mortality inequalities over two decades: register based study of European countries. BMJ.

[47] Noh H, Kim KS (2015). Revisiting the ‘feminisation of poverty’ in Korea: focused on time use and time poverty. Asia Pac J Soc Work Dev.

[48] Noh H, Kim YM (2020). Long-term trends and factors influencing the feminization of poverty. Issues Fem.

[49] Lee SR, Baek HY (2008). Regional variations of poverty in Korea: how are capital and metropolitan area different from non-capital and non-metropolitan area?. Korean J Soc Welf.

[50] GBD 2016 Causes of Death Collaborators (2017). Global, regional, and national age-sex specific mortality for 264 causes of death, 1980-2016: a systematic analysis for the Global Burden of Disease Study 2016. Lancet.

