# Supplementary material for: Trends and disparities in avoidable, treatable, and preventable mortalities in South Korea, 2001-2020: comparison of capital and non-capital areas
Source: Epidemiol Health. 2022 Aug 16;44:e2022067. doi: 10.4178/epih.e2022067 (PMC9754920; doi:10.4178/epih.e2022067)
Supplement: Supplementary Material 1. — Table S1. Definition of avoidable, treatable, and preventable deaths jointly developed by the OECD/Eurostat [1] Table S2. Changes in age-standardized mortality rates from avoidable, treatable, and preventable causes before and after 2009 by sex and region in South Korea Table S3. Correlation coefficients between the Gini coefficients of regional gross disposa-ble income per capita and disparities in avoidable, treatable, and preventable mortalities across areas in South Korea, 2001-2020 [file epih-44-e2022067-suppl1.docx]

Supplementary Table S1. Definition of avoidable, treatable, and preventable deaths jointly developed by the OECD/Eurostat [1]

| Group | Causes of deaths | | Preventable mortality | Treatable mortality | ICD-10 dode |
| --- | --- | --- | --- | --- | --- |
| Infectious diseases | Intestinal diseases | | x |  | A00-A09 |
|  | Diphtheria, tetanus, poliomyelitis | | x |  | A35, A36, A80 |
|  | Whooping cough | | x |  | A37 |
|  | Meningococcal infection | | x |  | A39 |
|  | Sepsis due to streptococcus pneumonia and sepsis due to haemophilus influenza | | x |  | A40.3, , A41.3 |
|  | Haemophilus influenza infections | | x |  | A49.2 |
|  | Sexually transmitted infections (except HIV/AIDS) | | x |  | A50-A60, A63, A64 |
|  | Varicella | | x |  | B01 |
|  | Measles | | x |  | B05 |
|  | Rubella | | x |  | B06 |
|  | Viral Hepatitis | | x |  | B15-B19 |
|  | HIV/AIDS | | x |  | B20-B24 |
|  | Malaria | | x |  | B50-B54 |
|  | Haemophilus and pneumococcal meningitis | | x |  | G00.0, G00.1 |
|  | Tuberculosis | | x (50%) | x (50%) | A15-A19, B90, J65 |
|  | Scarlet fever | |  | x | A38 |
|  | Sepsis | |  | x | A40 (excl. A40.3), A41 (excl. A41.3) |
|  | Cellulitis | |  | x | A46, L03 |
|  | Legionnaires disease | |  | x | A48.1 |
|  | Streptococcal and enterococci infection | |  | x | A49.1 |
|  | Other meningitis | |  | x | G00.2, G00.3, G00.8, G00.9 |
|  | Meningitis due to other and unspecified causes | |  | x | G03 |
|  | COVID-19 | | x |  | U07.1- U07.2 |
| Cancer | Lip, oral cavity and pharynx cancer | | x |  | C00-C14 |
|  | Esophageal cancer | | x |  | C15 |
|  | Stomach cancer | | x |  | C16 |
|  | Liver cancer | | x |  | C22 |
|  | Lung cancer | | x |  | C33-C34 |
|  | Mesothelioma | | x |  | C45 |
|  | Skin (melanoma) cancer | | x |  | C43 |
|  | Bladder cancer | | x |  | C67 |
|  | Cervical cancer | | x (50%) | x (50%) | C53 |
|  | Colorectal cancer | |  | x | C18-C21 |
|  | Breast cancer (female only) | |  | x | C50 |
|  | Uterus cancer | |  | x | C54,C55 |
|  | Testicular cancer | |  | x | C62 |
|  | Thyroid cancer | |  | x | C73 |
|  | Hodgkin's disease | |  | x | C81 |
|  | Lymphoid leukemia | |  | x | C91.0, C91.1 |
|  | Benign neoplasm | |  | x | D10-D36 |
| Endocrine and metabolic diseases | Nutritional deficiency anemia | | x |  | D50-D53 |
|  | Diabetes mellitus | | x (50%) | x (50%) | E10-E14 |
|  | Thyroid disorders | |  | x | E00-E07 |
|  | Adrenal disorders | |  | x | E24-E25 (except E24.4), E27 |
| Diseases of the nervous system | Epilepsy | |  | x | G40,G41 |
| Diseases of the circulatory system | Aortic aneurysm |  | x (50%) | x (50%) | I71 |
|  | Hypertensive diseases | | x (50%) | x (50%) | I10-I13, I15 |
|  | Ischemic heart diseases | | x (50%) | x (50%) | I20-I25 |
|  | Cerebrovascular diseases | | x (50%) | x (50%) | I60-I69 |
|  | Other atherosclerosis | | x (50%) | x (50%) | I70, I73.9 |
|  | Rheumatic and other heart disease | |  | x | I00-I09 |
|  | Venous thromboembolism | |  | x | I26, I80, I82.9 |
| Diseases of the respiratory system | Influenza | | x |  | J09-J11 |
|  | Pneumonia due to Streptococcus pneumonia or Haemophilus influenza | | x |  | J13-J14 |
|  | Chronic lower respiratory diseases | | x |  | J40-J44 |
|  | Lung diseases due to external agents | | x |  | J60-J64, J66-J70, J82, J92 |
|  | Upper respiratory infections | |  | x | J00-J06, J30-J39 |
|  | Pneumonia, not elsewhere classified or organism unspecified | |  | x | J12, J15, J16- J18 |
|  | Acute lower respiratory infections | |  | x | J20-J22 |
|  | Asthma and bronchiectasis | |  | x | J45-J47 |
|  | Adult respiratory distress syndrome | |  | x | J80 |
|  | Pulmonary edema | |  | x | J81 |
|  | Abscess of lung and mediastinum pyothorax | |  | x | J85, J86 |
|  | Other pleural disorders | |  | x | J90, J93, J94 |
| Diseases of the digestive system | Gastric and duodenal ulcer | |  | x | K25-K28 |
|  | Appendicitis | |  | x | K35-K38 |
|  | Abdominal hernia | |  | x | K40-K46 |
|  | Cholelithiasis and cholecystitis | |  | x | K80-K81 |
|  | Other diseases of gallbladder or biliary tract | |  | x | K82-K83 |
|  | Acute pancreatitis | |  | x | K85.0,1,3,8,9 |
|  | Other diseases of pancreas | |  | x | K86.1,2,3,8,9 |
| Diseases of the genitourinary system | Nephritis and nephrosis | |  | x | N00-N07 |
|  | Obstructive uropathy | |  | x | N13,N20-N21, N35 |
|  | Renal failure | |  | x | N17-N19 |
|  | Renal colic | |  | x | N23 |
|  | Disorders resulting from renal tubular dysfunction | |  | x | N25 |
|  | Unspecified contracted kidney, small kidney of unknown cause | |  | x | N26-N27 |
|  | Inflammatory diseases of genitourinary system | |  | x | N34.1,N70- N73,N75.0,N75.1,N76.4,6 |
|  | Prostatic hyperplasia | |  | x | N40 |
| Pregnancy, childbirth and perinatal period | Tetanus neonatorum | | x |  | A33 |
|  | Obstetrical tetanus | | x |  | A34 |
|  | Pregnancy, childbirth and the puerperium | |  | x | O00-O99 |
|  | Certain conditions originating in the perinatal period | |  | x | P00-P96 |
| Congenital malformations | Certain congenital malformations (neural tube defects) | | x |  | Q00, Q01, Q05 |
|  | Congenital malformations of the circulatory system (heart defects) | |  | x | Q20-Q28 |
| Adverse effects of medical and surgical care | Drugs, medicaments and biological substances causing adverse effects in therapeutic use | |  | x | Y40-Y59 |
|  | Misadventures to patients during surgical and medical care | |  | x | Y60-Y69,Y83-Y84 |
|  | Medical devices associated with adverse incidents in diagnostic and therapeutic use | |  | x | Y70–Y82 |
| Injuries | Transport accidents | | x |  | V01-V99 |
|  | Accidental injuries | | x |  | W00-X39, X46-X59 |
|  | Intentional self-harm | | x |  | X66-X84 |
|  | Event of undetermined intent | | x |  | Y16-Y34 |
|  | Assault | | x |  | X86-Y09 |
| Alcohol- related and drug-related deaths | Alcohol-related deaths | Alcohol- specific disorders and poisonings | x |  | E24.4, F10, G31.2, G62.1, G72.1, I42.6, K29.2, K70, K85.2, K86.0, Q86.0, R78.0, X45, X65, Y15 |
|  |  | Other alcohol-related disorders | x |  | K73, K74.0-K74.2, K74.6 |
|  | Drug-related deaths | Drug disorders and poisonings | x |  | F11-F16, F18-F19, X40- X44, X85, Y10-Y14 |
|  |  | Intentional self-poisoning by drugs | x |  | X60-X64 |

Age threshold: 0-74 years old

Table S2. Changes in age-standardized mortality rates from avoidable, treatable, and preventable causes before and after 2009 by sex and region in South Korea

| Mortality | Sex | Nationwide |  | Seoul Capital Area | | Non-Seoul-Capital metropolitan area | | Non-Seoul-Capital non-metropolitan area | |
| --- | --- | --- | --- | --- | --- | --- | --- | --- | --- |
|  |  | Periods | Annual percent change (95% CI) | Periods | Annual percent change (95% CI) | Periods | Annual percent change (95% CI) | Periods | Annual percent change (95% CI) |
| Avoidable mortality | Total | 2001-2009 | -4.8 (-5.6, -3.9) | 2001-2009 | -4.9 (-6.1, -3.8) | 2001-2009 | -4.5 (-5.3, -3.7) | 2001-2009 | -4.7 (-5.6, -3.8) |
|  |  | 2009-2020 | -4.5 (-4.9, -4.1) | 2009-2020 | -4.2 (-4.8, -3.7) | 2009-2020 | -4.6 (-5.0, -4.2) | 2009-2020 | -4.7 (-4.9, -4.6) |
|  | Men | 2001-2009 | -4.9 (-5.6, -4.2) | 2001-2009 | -5.0 (-5.9, -4.0) | 2001-2009 | -4.5 (-5.5, -3.4) | 2001-2009 | -4.9 (-6.0, -3.8) |
|  |  | 2009-2020 | -4.8 (-4.9, -4.7) | 2009-2020 | -4.4 (-4.5, -4.2) | 2009-2020 | -4.7 (-5.1, -4.2) | 2009-2020 | -5.0 (-5.2, -4.9) |
|  | Women | 2001-2009 | -5.2 (-6.6, -3.9) | 2001-2009 | -5.4 (-6.7, -4.1) | 2001-2009 | -5.4 (-5.5, -5.3) | 2001-2009 | -5.1 (-6.7, -3.4) |
|  |  | 2009-2020 | -4.6 (-5.3, -3.8) | 2009-2020 | -4.4 (-5.3, -3.5) | 2009-2020 | -4.8 (-5.3, -4.3) | 2009-2020 | -4.9 (-5.1, -4.7) |
| Treatable mortality | Total | 2001-2009 | -6.1 (-6.3, -5.9) | 2001-2009 | -6.5 (-7.6, -5.4) | 2001-2009 | -5.7 (-6.0, -5.4) | 2001-2009 | -5.7 (-6.0, -5.3) |
|  |  | 2009-2020 | -3.7 (-3.9, -3.6) | 2009-2020 | -3.6 (-3.8, -3.5) | 2009-2020 | -4.3 (-4.5, -4.1) | 2009-2020 | -3.5 (-3.7, -3.2) |
|  | Men | 2001-2009 | -6.2 (-6.4, -5.9) | 2001-2009 | -6.9 (-7.2, -6.6) | 2001-2009 | -5.7 (-6.1, -5.4) | 2001-2009 | -5.6 (-6.1, -5.2) |
|  |  | 2009-2020 | -3.7 (-3.8, -3.5) | 2009-2020 | -3.5 (-3.7, -3.3) | 2009-2020 | -4.3 (-4.5, -4.0) | 2009-2020 | -3.7 (-4.0, -3.5) |
|  | Women | 2001-2009 | -6.3 (-6.6, -5.9) | 2001-2009 | -6.8 (-7.2, -6.3) | 2001-2009 | -5.9 (-6.1, -5.7) | 2001-2009 | -5.9 (-6.4, -5.5) |
|  |  | 2009-2020 | -4.0 (-4.3, -3.8) | 2009-2020 | -4.1 (-4.4, -3.8) | 2009-2020 | -4.8 (-5.4, -4.1) | 2009-2020 | -3.7 (-4.0, -3.4) |
| Preventable mortality | Total | 2001-2009 | -4.3 (-4.9, -3.7) | 2001-2009 | -4.7 (-4.8, -4.5) | 2001-2009 | -4.1 (-5.3, -2.8) | 2001-2009 | -4.3 (-5.6, -3.1) |
|  |  | 2009-2020 | -5.0 (-5.2, -4.8) | 2009-2020 | -4.7 (-4.8, -4.5) | 2009-2020 | -5.0 (-5.1, -4.8) | 2009-2020 | -5.3 (-5.5, -5.0) |
|  | Men | 2001-2009 | -4.5 (-5.1, -3.9) | 2001-2009 | -4.4 (-5.4, -3.4) | 2001-2009 | -4.2 (-4.9, -3.4) | 2001-2009 | -4.6 (-5.9, -3.4) |
|  |  | 2009-2020 | -5.1 (-5.3, -5.0) | 2009-2020 | -4.7 (-4.9, -4.4) | 2009-2020 | -5.0 (-5.2, -4.9) | 2009-2020 | -5.6 (-5.8, -5.3) |
|  | Women | 2001-2009 | -4.9 (-5.7, -4.0) | 2001-2009 | -5.4 (-5.6, -5.2) | 2001-2009 | -4.8 (-5.8, -3.9) | 2001-2009 | -5.4 (-5.6, -5.1) |
|  |  | 2009-2020 | -5.0 (-5.6, -4.4) | 2009-2020 | -4.6 (-5.8, -3.4) | 2009-2020 | -4.8 (-6.1, -3.5) | 2009-2020 | -5.4 (-5.6, -5.1) |

Abbreviations: CI, confidence interval.

Table S3. Correlation coefficients between the Gini coefficients of regional gross disposable income per capita and disparities in avoidable, treatable, and preventable mortalities across areas in South Korea, 2001-2020

| Mortality | Disparity measure | | Total |  | Men |  | Women |  |
| --- | --- | --- | --- | --- | --- | --- | --- | --- |
|  |  |  | Pearson correlation coefficient | Spearman correlation coefficient | Pearson correlation coefficient | Spearman correlation coefficient | Pearson correlation coefficient | Spearman correlation coefficient |
| Avoidable mortality | Absolute measure | RD | **0.711** | **0.689** | **0.711** | **0.720** | **0.681** | **0.611** |
|  |  | BGV | **0.690** | **0.686** | **0.685** | **0.720** | **0.653** | **0.656** |
|  |  | eACI | **0.710** | **0.686** | **0.709** | **0.720** | **0.683** | **0.632** |
|  |  | SII | **0.711** | **0.678** | **0.710** | **0.720** | **0.683** | **0.632** |
|  | Relative measure | RR | **0.599** | **0.549** | **0.741** | **0.705** | -0.119 | -0.217 |
|  |  | IDisp | **0.520** | **0.454** | **0.663** | **0.600** | 0.040 | -0.135 |
|  |  | MLD | **0.578** | **0.535** | **0.726** | **0.663** | -0.058 | -0.168 |
|  |  | T | **0.578** | **0.535** | **0.729** | **0.663** | -0.063 | -0.171 |
|  |  | eRCI | **0.607** | **0.546** | **0.736** | **0.690** | -0.064 | -0.185 |
|  |  | RII | **0.575** | **0.540** | **0.730** | **0.639** | -0.099 | -0.238 |
|  |  | KMI | **0.568** | **0.540** | **0.726** | **0.639** | -0.106 | -0.238 |
| Treatable mortality | Absolute measure | RD | **0.509** | **0.537** | **0.595** | **0.562** | 0.290 | 0.221 |
|  |  | BGV | 0.423 | 0.403 | **0.563** | **0.579** | 0.187 | 0.059 |
|  |  | eACI | 0.112 | 0.062 | **0.501** | **0.462** | -0.284 | -0.388 |
|  |  | SII | 0.096 | 0.032 | **0.489** | **0.462** | -0.291 | -0.392 |
|  | Relative measure | RR | -0.085 | -0.044 | 0.111 | 0.018 | -0.328 | -0.356 |
|  |  | IDisp | -0.168 | -0.203 | 0.042 | -0.080 | -0.328 | -0.421 |
|  |  | MLD | -0.172 | -0.209 | 0.047 | -0.114 | -0.334 | **-0.477** |
|  |  | T | -0.171 | -0.209 | 0.048 | -0.114 | -0.334 | **-0.477** |
|  |  | eRCI | -0.383 | -0.411 | -0.170 | -0.164 | **-0.495** | **-0.550** |
|  |  | RII | -0.388 | -0.417 | -0.178 | -0.164 | **-0.498** | **-0.550** |
|  |  | KMI | -0.383 | -0.417 | -0.166 | -0.164 | **-0.492** | **-0.550** |
| Preventable mortality | Absolute measure | RD | **0.694** | **0.699** | **0.695** | **0.687** | **0.667** | **0.632** |
|  |  | BGV | **0.666** | **0.695** | **0.664** | **0.687** | **0.639** | **0.650** |
|  |  | eACI | **0.692** | **0.690** | **0.693** | **0.687** | **0.666** | **0.632** |
|  |  | SII | **0.693** | **0.701** | **0.694** | **0.699** | **0.667** | **0.632** |
|  | Relative measure | RR | **0.730** | **0.678** | **0.750** | **0.704** | 0.426 | 0.278 |
|  |  | IDisp | **0.649** | **0.618** | **0.692** | **0.654** | 0.390 | 0.314 |
|  |  | MLD | **0.724** | **0.665** | **0.749** | **0.704** | 0.403 | 0.301 |
|  |  | T | **0.725** | **0.665** | **0.750** | **0.704** | 0.401 | 0.301 |
|  |  | eRCI | **0.724** | **0.678** | **0.745** | **0.689** | 0.439 | 0.287 |
|  |  | RII | **0.720** | **0.665** | **0.745** | **0.704** | 0.410 | 0.283 |
|  |  | KMI | **0.719** | **0.665** | **0.746** | **0.704** | 0.400 | 0.283 |

Correlation coefficients in bolds are statistically significant at p <0.05. Abbreviations: RD, range difference; BGV, between group variance; eACI, extended absolute concentration index; SII, slope index of inequality; RR, range ratio; IDisp, index of disparity; MLD, mean log deviation; T, Theil index; eRCI, extended relative concentration index; RII, relative index of inequality; KMI, Kunst-Mackenbach relative index.

**References**

1.OECD, Eurostat. Avoidable mortality: OECD/Eurostat lists of preventable and treatable causes of death [cited 2022 January 5]. Available from: <https://www.oecd.org/health/health-systems/Avoidable-mortality-2019-Joint-OECD-Eurostat-List-preventable-treatable-causes-of-death.pdf>.
