# Supplementary material for: Trends and disparities in avoidable, treatable, and preventable mortalities in South Korea, 2001-2020: comparison of capital and non-capital areas
Source: Epidemiol Health. 2022 Aug 16;44:e2022067. doi: 10.4178/epih.e2022067 (PMC9754920; doi:10.4178/epih.e2022067)
Supplement: Supplementary Material 2. — Appendix A: definition of health disparity measures [1-3] Appendix B: joinpoint regression model [4-8] [file epih-44-e2022067-suppl2.docx]

**Supplementary Appendix A: definition of health disparity measures [1-3]**

1. Range difference (RD)

$$\hat{RD}=y_{max}-y_{min}$$

$y_{max}$: Health status of the best group

$y_{min}$: Health status of the worst group

2. Between-group variance (BGV)

$\hat{BGV}=\sum_{j=1}^{J} {p_{j}(y_{j}-\mu)}^{2}$

$y_{j}$: Average health status of group j

$p_{j}$: Population size of group j

$\mu$: Average health status of the population, $\mu=\sum_{j=1}^{J} p_{j}y_{j}$

3. Extended absolute concentration index (eACI)

$$\hat{eACI}=\mu(\hat{eRCI})=\nu\mu\sum_{j=1}^{J} {p_{j}(1-R_{j})}^{\nu-1}-\nu\sum_{j=1}^{J} {p_{j}y_{j}(1-R_{j})}^{\nu-1}$$

$\nu$: Aversion parameter (>0)

$y_{j}$: Average health status of group j

$p_{j}$: Population size of group j

$\mu$: Average health status of the population, $\mu=\sum_{j=1}^{J} p_{j}y_{j}$

$R_{j}$: Relative rank of the j^th^ socioeconomic group, $R_{j}=\sum_{\gamma=1}^{j-1} p_{\gamma}-0.5p_{j}$

4. Slope index of inequality (SII)

$$y_{j}=\beta_{0}+\beta_{1}R_{j}$$

$\beta_{0}$: Estimated health status of a hypothetical person at the bottom of social hierarchy

$\beta_{1}$: Difference in average health status between the hypothetical person at the bottom of social hierarchy and the hypothetical person at the top

$$\hat{SII}=\hat{\beta_{1}}=\frac{\sum_{j=1}^{J} p_{j}R_{j}(y_{j}-\mu)}{\sum_{j=1}^{J} p_{j}R_{j}^{2}-{(\sum_{j=1}^{J} p_{j}R_{j})}^{2}}$$

$y_{j}$: Average health status of group j

$p_{j}$: Population size of group j

$\mu$: Average health status of the population, $\mu=\sum_{j=1}^{J} p_{j}y_{j}$

$R_{j}$: Relative rank of the j^th^ socioeconomic group, $R_{j}=\sum_{\gamma=1}^{j-1} p_{\gamma}-0.5p_{j}$

5. Range ratio (RR)

$$\hat{RR}=\frac{y_{max}}{y_{min}}$$

$y_{max}$: Health status of the best group

$y_{min}$: Health status of the worst group

6. Index of disparity (IDisp)

$$\hat{IDisp}=100\times\frac{1}{J-1}\sum_{j=1}^{J} , j\neq ref\frac{\left| y_{j}-y_{ref} \right|}{y_{ref}}$$

$y_{j}$: Average health status of group j

$y_{ref}$: Health status indicator in the reference group

7. Mean log deviation (MLD)

$$\hat{MLD}=-\sum_{j=1}^{J} p_{j}\ln\left( \frac{y_{j}}{\mu} \right)=\ln\left( \mu\right)-\sum_{j=1}^{J} p_{j}ln(y_{j})$$

$y_{j}$: Average health status of group j

$p_{j}$: Population size of group j

$\mu$: Average health status of the population, $\mu=\sum_{j=1}^{J} p_{j}y_{j}$

8. Extended relative concentration index (eRCI)

$$\hat{eRCI}= \nu\sum_{j=1}^{J} {p_{j}(1-R_{j})}^{\nu-1}-\frac{\nu}{\mu}\sum_{j=1}^{J} {p_{j}y_{j}(1-R_{j})}^{\nu-1}$$

$\nu$: Aversion parameter (>0)

$y_{j}$: Average health status of group j

$p_{j}$: Population size of group j

$\mu$: Average health status of the population, $\mu=\sum_{j=1}^{J} p_{j}y_{j}$

$R_{j}$: Relative rank of the j^th^ socioeconomic group, $R_{j}=\sum_{\gamma=1}^{j-1} p_{\gamma}-0.5p_{j}$

9. Theil index (T)

$$\hat{T}=\sum_{j=1}^{J} p_{j}Z_{j}ln(Z_{j})$$

$p_{j}$: Population size of group j

$Z_{j}=\frac{y_{j}}{\mu}$: Ratio of the health status of the j^th^ socioeconomic group ($y_{j}$) relative to the average health status of the population ($\mu$), $\mu=\sum_{j=1}^{J} p_{j}y_{j}$

10. Relative index of inequality (RII)

$$\hat{RII}=\frac{SII}{\mu}=\frac{\hat{\beta_{1}}}{\mu}=\frac{1}{\sum_{j=1}^{J} p_{j}R_{j}^{2}-{(\sum_{j=1}^{J} p_{j}R_{j})}^{2}}\left[ \frac{\sum_{j=1}^{J} p_{j}R_{j}y_{j}}{\sum_{j=1}^{J} p_{j}y_{j}}-\sum_{j=1}^{J} p_{j}R_{j} \right]$$

$y_{j}$: Average health status of group j

$p_{j}$: Population size of group j

$\mu$: Average health status of the population, $\mu=\sum_{j=1}^{J} p_{j}y_{j}$

$R_{j}$: Relative rank of the j^th^ socioeconomic group, $R_{j}=\sum_{\gamma=1}^{j-1} p_{\gamma}-0.5p_{j}$

11. Kunst Mackenbach relative index (KMI)

$$\hat{KMI}=\frac{\hat{\beta_{0}}}{\hat{\beta_{0}}+\hat{\beta_{1}}}=\frac{\hat{\beta_{0}}}{\hat{\beta_{0}}+SII}$$

$\hat{\beta_{0}}$: Estimated health status of a hypothetical person at the bottom of social hierarchy

$\hat{\beta_{1}}$: Difference in average health status between the hypothetical person at the bottom of social hierarchy and the hypothetical person at the top

$R_{j}$: Relative rank of the j^th^ socioeconomic group, $R_{j}=\sum_{\gamma=1}^{j-1} p_{\gamma}-0.5p_{j}$

**Supplementary Appendix B: joinpoint regression model [4-8]**

Joinpoint regression analysis is widely used to identify the time points when the trends of rates, proportions, or any kind of measures change statistically significantly [4-8]. For the observations $\left( x_{1},y_{1} \right),\ldots,\left( x_{n},y_{n} \right)$, where $x_{1}<x_{2}<...<x_{n}$ denotes the time variable (i.e., year) and $y_{i} (i=1,\ldots,n)$ denotes the outcome variable (i.e., age-standardized mortality rates from avoidable, treatable, and preventable causes by sex and region, and absolute and relative disparities between areas), the joinpoint regression model is as follows [4-8]:

$\ln\left( y_{i} \right)=E\left[ y_{i} | x_{i} \right]+\varepsilon_{i}$, where $\varepsilon_{i}$ is the residual for the *i*^th^ time, $E\left[ y_{i} | x_{i} \right]$ denotes the regression mean which is defined as a series of $(n+1)$ linear segments over the period $[a,b]$: $E\left[ y_{i} | x_{i} \right]=\beta_{0}+\beta_{1}x_{i}+\delta_{i}{(x_{i}-\tau_{1})}^{+}+...+\delta_{n}{(x_{i}-\tau_{n})}^{+}$, where ${(x_{i}-\tau_{k})}^{+}=\left( x_{i}-\tau_{k} \right)$ for $x_{i}>\tau_{k}$ and 0 otherwise [4-8]. $\delta_{n}$ denotes the difference between slopes of the $(k+1)$^th^ and $k$^th^ segments [4-8]. $\tau_{k}, k=1,2,\ldots,n, n<N$ represents the $k$th unknown joinpoint or time point at which the trend changed statistically significantly with $\tau_{0}=a$ and $\tau_{n+1}=b$ [4-8]. $\tau_{l}<...<\tau_{k}$ are the joinpoints [4-8]. The annual percent change between two adjacent joinpoints $\tau_{k}$ and $\tau_{k+1}$ was calculated as $(e^{(\beta_{1}+\delta_{1}+\ldots\delta_{k})}-1)\times100$ [4-8].

The parameters were set to specify joinpoint regression model as follows: standard errors calculated from the study data were used as random errors and autocorrelation of random errors was considered. The grid search method, which enables the joinpoints to occur precisely at the observed years, was selected to find the position of the joinpoints [4-8]. The optimal number of joinpoints was determined by sequentially performing Monte Carlo permutation test with 4499 iterations. Each of the permutations tests the null hypothesis $H_{0}$: number of joinpoints $=k_{a}$ against the alternative $H_{a}$: number of joinpoints ${=k}_{b}$, where $k_{a}<k_{b}$ [4-8]. It is conducted beginning with $k_{a}=MIN or minimum number of joinpoi\mathrm{nts}$ and up to $k_{b}=MAX or \mathrm{maximum} number of joinpoi\mathrm{nts}$ [4-8]. Minimum and maximum numbers of joinpoints were set as 0 and 3, respectively. Monte Carlo simulation produces a p-value for each hypothesis test, and when the null hypothesis is rejected, the number of joinpoints in the null model is incremented by one, whereas when the null hypothesis is accepted, the number of joinpoints in the alternative model is decremented by one [4-8]. This process continues until no more joinpoints need to be added to the model [4-8].

**References**

1. National Cancer Institute. Health Disparities Calculator, version 2.0.0: Surveillance Research Program and Healthcare Delivery Research Program, National Cancer Institute; 2019 [cited 2022 March 18]. Available from: <https://seer.cancer.gov/hdcalc/>.

2. Harper S, Lynch J. Selected comparisons of measures of health disparities: a review using databases relevant to Healthy People 2010 cancer-related objectives. Bethesda, MD: National Cancer Institute; 2007.

3. Harper S, Lynch J. Methods for measuring cancer disparities: using data relevant to Healthy People 2010 cancer-related objectives. Bethesda, MD: National Cancer Institute; 2005.

4. National Cancer Institute. Joinpoint Regression Program, version 4.9.1.0: Statistical Research and Applications Branch, National Cancer Institute; 2022 [cited 2022 April 14]. Available from: <https://surveillance.cancer.gov/joinpoint/>.

5. Bandi P, Silver D, Mijanovich T, Macinko J. Temporal trends in motor vehicle fatalities in the United States, 1968 to 2010 - a joinpoint regression analysis. Inj Epidemiol 2015;2(1):4.

6. Rea F, Pagan E, Compagnoni MM, Cantarutti A, Pugni P, Bagnardi V, et al. Joinpoint regression analysis with time-on-study as time-scale. Application to three Italian population-based cohort studies. Epidemiol Biostat Public Health 2017;14(3):e12616.

7. Dragomirescu I, Llorca J, Gómez-Acebo I, Dierssen-Sotos T. A join point regression analysis of trends in mortality due to osteoporosis in Spain. Sci Rep 2019;9(1):4264.

8. Goovaerts P, Xiao H. Geographical, temporal and racial disparities in late-stage prostate cancer incidence across Florida: a multiscale joinpoint regression analysis. Int J Health Geogr 2011;10:63.
